# Role of positron emission tomography-computed tomography in bronchial mucoepidermoid carcinomas: a case series and review of the literature

**DOI:** 10.1186/1752-1947-4-277

**Published:** 2010-08-19

**Authors:** Tarun Jindal, Arvind Kumar, Rakesh Kumar, Roman Dutta, Monika Meena

**Affiliations:** 1 Department of Surgical Disciplines, All India Institute of Medical Sciences, New Delhi-11029, India; 2 Department of Nuclear Medicine, All India Institute of Medical Sciences, New Delhi-11029, India

## Abstract

**Introduction:**

Mucoepidermoid carcinoma of the tracheobronchial tree is rare. Such tumors usually present with signs and symptoms of bronchial obstruction. Histologically, they can be classified as high-grade or low-grade tumors. Experience of imaging these tumors with ^18^fluorodeoxyglucose positron emission tomography-computed tomography (^18^F-FDG PET-CT) is limited. We present three cases of this rare tumor, describe the functional imaging results, and review the available literature.

**Case presentation:**

Three Caucasian patients, two men (21 and 24 years of age) and one woman (14 years of age), with bronchial masses were evaluated by us. All three patients were symptomatic, and underwent a thorough clinical examination, bronchoscopy and biopsy, plain computed tomography, ^18^F-FDG PET-CT and ^68^Gallium 1,4,7,10-Tetraazacyclododecane-N^I^,N^II^,N^III^,N^IIII^,- tetra acetic acid (D) - Phel^1^-Tyr^3^-octreotide positron emission tomography-computed tomography (^68^Ga-DOTATOC PET-CT). ^18^F-FDG PET-CT revealed mild uptake in all three patients, whereas ^68^Ga-DOTATOC PET-CT revealed no significant uptake in any patient, making carcinoid tumor unlikely. Results of histopathological examination were consistent with low-grade mucoepidermoid carcinoma in all patients.

**Conclusion:**

Our study reveals that functional imaging may be helpful in the initial investigation of patients with mucoepidermoid carcinoma. ^18^F-FDG PET-CT may have a prognostic relevance by predicting the histopathologic differentiation of the tumor.

## Introduction

Mucoepidermoid carcinoma (MEC) of the tracheal-bronchial tree is rare, comprising only 0.1% to 0.2% of primary lung malignancies [1]. It is believed to originate from the minor salivary glands lining the tracheal-bronchial tree. Although considered an indolent tumor, local invasion and lymph node metastases may occur. Computed tomography (CT) usually reveals a solitary nodule or an endobronchial mass with or without post-obstructive pneumonia or atelectasis [2]. Recently, ^18^fluorodeoxyglucose (^18^F-FDG) positron emission tomography (PET)-CT has been reported to be useful in lung cancers and carcinoids. However, the literature on PET-CT findings in MECs is scanty. We present three cases of this rare tumor with the FDG uptake patterns, and review the available literature.

## Case presentation

This was an investigative protocol, which was approved by the ethics committee of our institution, and informed consent was gained for each patient.

All patients presenting with tracheal-bronchial tumors underwent ^18^F-FDG PET-CT and ^68^Gallium 1,4,7,10-Tetraazacyclododecane-N^I^,N^II^,N^III^,N^IIII^,- tetra acetic acid (D) - Phel^1^-Tyr^3^-octreotide (^8^Ga-DOTATOC) PET-CT using a dedicated PET-CT scanner (Biograph 64; Siemens Medical Solutions Inc, Mountain View, CA, USA) to assess the diagnostic value of these methods in evaluating bronchial tumors.

We present three such cases, for which the histologic diagnosis was MEC, and retrospectively analyze their PET-CT findings and histopathologic grading. Their clinical parameters and other details are given in Table 1.

**Table 1:** Patient characteristics and other details

Case number	Age/sex	Symptoms	CT findings	Bronchoscopy	Bronchoscopic biopsy	FDG PET-CT scan	DOTATOC PET-CT scan	Operative procedure	Final diagnosis
**1**	14/F	C, D × 1 year	27 × 16 mm mass occluding the left main bronchus.	Infiltrative growth occluding the left main bronchus	Inconclusive	Uptake positive (SUVmax 4.4) (Figure 1)	No significant uptake (Figure 2)	Sleeve resection of left main bronchus	Low-grade MEC
**2**	21/M	C, D × 1 year	10 × 10 mm mass in the right main bronchus	Polypoidal mass in the right main bronchus	Low-grade MEC	Uptake positive (SUVmax 3.2)	No significant uptake	No surgery yet	Low-grade MEC
**3**	24/M	C, H × 1 year	35 × 38 mm mass in right main bronchus going up to the carina	Polypoidal growth starting at carina and occluding right main bronchus	?Neuroendocrine tumor	Uptake positive (SUVmax 3.9)	No significant uptake	Right pneumonectomy with carinal resection	Low-grade MEC

C- Cough, D- Dyspnea, DOTATOC PET-CT (1,4,7,10-tetraazacyclododecane-N^I^,N^II^,N^III^,N^IIII^,- tetra acetic acid (D) - Phel^1^-Tyr^3^-octreotide positron emission tomography computed tomography), F- Female, FDG PET-CT- fluorodeoxyglucose positron emission tomography computed tomography, H-Haemoptysis, M- Male, MEC- mucoepidermoid carcinoma, SUVmax- maximum standardized uptake value.

### Case 1

**Figure 1:**
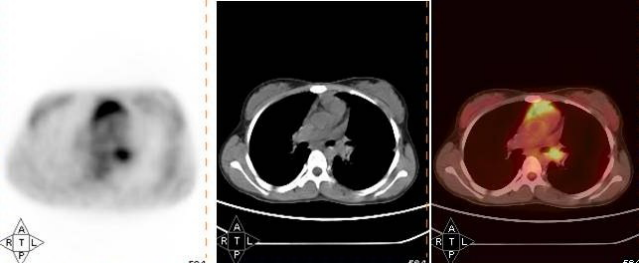
^**18**^**F fluorodeoxyglucose (FDG) findings in the histologically proved low grade left bronchial mucoepidermoid carcinoma**. Axial section of positron emission tomography (PET), computed tomography and PET-computed tomography images of ^18^F FDG scan showing mild FDG uptake in the histologically proved low grade left bronchial mucoepidermoid carcinoma (case 1).

**Figure 2:**
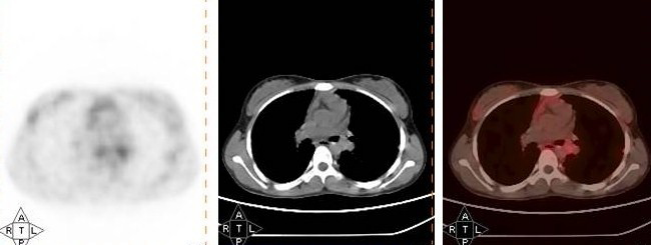
^**68**^**Gallium 1,4,7,10-Tetraazacyclododecane-N**^**I**^**,N**^**II**^**,N**^**III**^**,N**^**IIII**^**,- tetra acetic acid (D) - Phel**^**1**^**-Tyr**^**3**^**-octreotide (**^**68**^**Ga-DOTATOC) findings in the histologically proved low grade left bronchial mucoepidermoid carcinoma**. Axial section of positron emission tomography (PET), computed tomography and PET-computed tomography images of ^68^Ga DOTATOC scan showing no significant radiotracer uptake in the histologically proved low grade left bronchial mucoepidermoid carcinoma (case 1).

A 14-year-old Caucasian girl presented with a one-year history of cough and gradually progressive dyspnea. On clinical examination, decreased air entry was noted on the left side of the chest. Contrast enhanced CT (CECT) of the chest revealed a mass measuring 27 × 16 mm and occluding the left main bronchus. On bronchoscopy, the mass was seen to be occluding the left main bronchus. Results of a bronchoscopic biopsy were inconclusive. The patient underwent a ^18^F-FDG PET-CT scan (Figure 1) which revealed mild uptake in the tumor (maximum standardized uptake value (SUVmax) 4.4), whereas ^68^Ga-DOTATOC PET-CT revealed no significant uptake (Figure 2). The patient underwent a sleeve resection of the tumor, and had an uneventful recovery. Histopathologic examination revealed features of low-grade MEC.

### Case 2

A 21-year-old Caucasian man presented with a one-year history of cough and dyspnea on exertion. CECT of the chest revealed a 10 × 10 mm mass in the right main bronchus. Bronchoscopic examination revealed a mass in the right main bronchus, which bled on contact. ^18^F-FDG PET-CT revealed slight uptake (SUVmax 3.2) in the tumor, whereas ^68^Ga-DOTATOC PET-CT revealed no significant uptake. Histological results of a biopsy taken from the mass were suggestive of low-grade MEC.

### Case 3

A 24-year-old Caucasian man presented with a one-year history of cough and haemoptysis. On clinical examination, decreased air entry was detected on the right side of the chest. CECT revealed a mass of 35 × 38 mm in the right main bronchus with collapse of the right lung. Bronchoscopic examination revealed a mass starting at the level of the carina, occluding the right main bronchus completely. Histopathological examination of a bronchoscopic biopsy of the mass was suggestive of a neuroendocrine tumor. ^18^F-FDG PET-CT revealed mild uptake in the tumor (SUVmax 3.9), whereas ^68^Ga-DOTATOC PET-CT revealed no significant uptake. The patient underwent a right pneumonectomy with resection of the carina. Histopathologic examination of the operative specimen revealed features of low-grade MEC.

## Discussion

MEC is an uncommon lesion accounting for under 1% of primary malignant bronchial tumors. Although generally indolent, local invasion and lymph node metastases may occur. The tumor generally affects patients aged over 30 years. Patients usually present with cough, haemoptysis, wheezing and recurrent pneumonia due to bronchial obstruction, but 9% to 28% of cases may be asymptomatic [2,3].

Histologically, MECs are composed of varying mixtures of mucus-secreting, columnar and goblet cells. They are classified as high-grade or low-grade based on histologic appearance (number of mitoses, nuclear pleomorphism and necrosis). The histopathologic grading also reflects the prognosis of these tumors [2].

On conventional radiology, MECs usually appear as oval or lobulated, slightly enhanced, endobronchial masses with occasional punctuate calcification. There may be post-obstructive pneumonia and/or peripheral atelectasis [3].

Functional imaging is emerging as a helpful tool for the evaluation of bronchopulmonary tumors. However, experience in MECs is limited, with 12 cases reported from six studies (summarized in Table 2) [2-7]. The range of SUVmax values on ^18^F-FDG PET-CT scan varies from zero to 6.2 for low-grade MECS and from 2.86 to 23.4 for high-grade MECs. We found uniformly slight uptake (low SUV) on ^18^F-FDG PET-CT in all three cases, all of which had a histopathologic diagnosis of low-grade MEC (Figure 1). Thus, our study suggests that the SUVmax on ^18^F-FDG PET-CT scan may be a predictor for histopathologic differentiation of MEC.

**Table 2:** Review of the literature of pulmonary mucoepidermoid carcinomas

Patient number	Reference	Number of cases	Grade		Range of SUVmax on FDG scan	
			
			Low	High	Low grade	High grade
1	Lee *et al. *[2]	1	1	-	6.2	-
**2**	Jeong *et al. *[3]	7	4	2	1.5 to 5.5	4.8 to 23.4

**3**	Ishizumi *et al. *[4]	1	1	-	3.63	-

**4**	Kinoshita *et al. *[5]	1	-	-	-	-

**5**	Yamada *et al. *[6]	1	-	1	-	2.86

**6**	Shim *et al. *[7]	1	-	-	No uptake	-

Bronchial carcinoids constitute a common differential diagnosis for MECs, both by structural radiology and by ^18^F-FDG PET-CT. In doubtful cases, such as those with an inconclusive preoperative biopsy, a ^68^Ga-DOTATOC PET-CT scan can be performed, results of which are usually positive for typical bronchial carcinoids and negative for MECs (Figure 2) [8].

## Conclusion

Although the overall reported experience is very limited, ^18^F-FDG PET-CT scan might be a useful method for assessing MECs. ^18^F-FDG PET-CT may also have a prognostic relevance by predicting the histopathologic differentiation of the tumor.

## Abbreviations

^18^F-FDG PET-CT: fluorodeoxyglucose positron emission tomography-computed tomography; ^68^Ga-DOTATOC PET-CT: ^68^gallium 1,4,7,10-tetraazacyclododecane-N^I^,N^II^,N^III^,N^IIII^,- tetra acetic acid (D)-Phel^1^-Tyr^3^-octreotide positron emission tomography-computed tomography; CECT: contrast enhanced computed tomography; MEC: mucoepidermoid carcinoma, SUVmax: standardized uptake value.

## Consent

Written informed consent was obtained from patients two, three and from patient one's father for publication of this case report and accompanying images. A copy of the written consent is available for review by the Editor-in-Chief of this journal.

## Competing interests

The authors declare that they have no competing interests.

## Authors' contributions

TJ conceived the study and made a major contribution in the compilation, analysis, literature review and formatting of the manuscript, AK had a major contribution in the analysis and editing, RK helped in the study design and data acquisition, RD contributed in data analysis, MM helped in the review of the literature. All the authors have read the final manuscript and have approved it.
